# Contrast enhanced mammography: focus on frequently encountered benign and malignant diagnoses

**DOI:** 10.1186/s40644-023-00526-1

**Published:** 2023-01-23

**Authors:** Mindy L. Yang, Chandni Bhimani, Robyn Roth, Pauline Germaine

**Affiliations:** 1 Department of Radiology, Cooper University Hospital, 1 Cooper Plaza, Camden, NJ 08103 USA; 2 Present address: SimonMed Imaging, 6900 E Camelback Road, Suite 700, Scottsdale, AZ 85251 USA; 3 Present address: Atlantic Medical Imaging, Bayport One Office Building, 8025 Black Horse Pike, Suite 300, West Atlantic City, NJ 08232 USA

**Keywords:** Contrast-enhanced mammography, Contrast mammography, Mammography

## Abstract

Contrast-enhanced mammography (CEM) is becoming a widely adopted modality in breast imaging over the past few decades and exponentially so over the last few years, with strong evidence of high diagnostic performance in cancer detection. Evidence is also growing indicating comparative performance of CEM to MRI in sensitivity with fewer false positive rates. As application of CEM ranges from potential use in screening dense breast populations to staging of known breast malignancy, increased familiarity with the modality and its implementation, and disease processes encountered becomes of great clinical significance. This review emphasizes expected normal findings on CEM followed by a focus on examples of the commonly encountered benign and malignant pathologies on CEM.

## Introduction

Contrast-enhanced mammography (CEM) has been gaining traction as a modality in breast imaging over the past few decades since its introduction to clinical use in 2003 [1, 2]. It combines full field digital mammography (FFDM), a standard screening test, with a dual energy technique, while utilizing iodinated contrast injection. Several studies have revealed encouraging results especially for the use of CEM in clarifying equivocal mammographic findings, detection of occult findings particularly in dense breasts, and assessment of disease extent as well as treatment response in known malignancy [3]. CEM diagnostic performance is higher than that of FFDM or FFDM with ultrasound [4–8]. Furthermore, over the past few years, studies comparing CEM to MRI indicate overall similar performance in sensitivity [9–12] and negative predictive values, with decreased false positive rates. With increasing utilization by practices worldwide and variable application in diverse diagnostic and potential screening settings, there remains a need to be familiar with the modality, it’s clinical implementation, and commonly encountered disease processes, both benign and malignant.

In this article, we focus on CEM appearance of commonly encountered benign and malignant pathologies, in addition to highlighting limitations of the modality. Utilizing adapted BIRADS descriptors for CEM interpretation, which have been shown by Berg et al. [13] to be comparable in consistency to the usage of BIRADS lexicons in other modalities, for the characterization of variable pathologies, will lead to improved standardization of CEM interpretation, reporting and application in continually expanding clinical scenarios.

## Technique

Software modification to standard mammography equipment is required to perform CEM. Appropriate training for radiologists and technologists is essential prior to implementation of CEM into clinical practice [14].

**Fig. 1:**
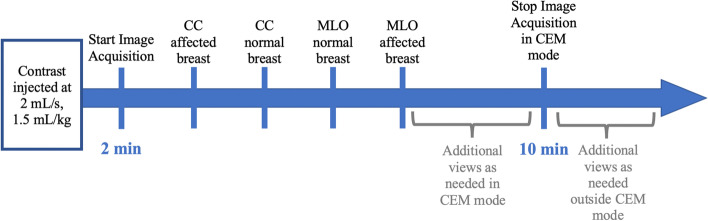
Current CEM protocol

Prior to acquiring images, peripheral access is obtained by a trained technologist or nurse, preferably with a 20-22-gauge needle. The protocol detailed below is currently utilized in our institution (Fig. 1). A dose of 1.5 mL/kg of iodinated contrast material (Isovue 370 at our institution) is calculated and administered intravenously utilizing a power injector at a rate of 2 mL/s. A tight delivery of contrast is achieved by administering a 20 mL saline bolus both prior to and following contrast injection to optimize image quality. Following a two-minute delay, image acquisition begins, with completion of image acquisition within 10 min of initiation of intravenous injection. The patient is monitored for a rare event of adverse reaction to the iodinated contrast material, which is essentially nonexistent, likely at similar rates to those reported for administration during CT examinations [15]. A kit of appropriate medications is available in the room for management of contrast reactions of various severities.

Image acquisition includes simultaneous full-field exposures obtained at high and low energies utilizing standard CC and MLO projections of each breast. Per institutional protocol, the CC projection on the side of interest is imaged first with the goal of capturing early arterial enhancement and to minimize false negative results from early wash out. The contralateral breast is then imaged in CC and MLO projections. The MLO projection of the side of interest is the last to be performed in an attempt to assess washout kinetics. Low energy (LE) mammograms are performed at the same peak kilovoltage (kVp) of 26-30 kVp, and with the same filtration as standard full field digital mammography [16–20]. The high energy (HE) acquisition is performed at a higher kV (45-49 kVp), taking into account the K edge of iodinated contrast, and with stronger filtration (copper) [16–20]. Subtraction/recombined images (RI) are automatically produced by the software. The LE image and RI are sent to PACS; the HE images are only utilized to obtain RI and thus are not directly viewed or interpreted.

**Fig. 2:**
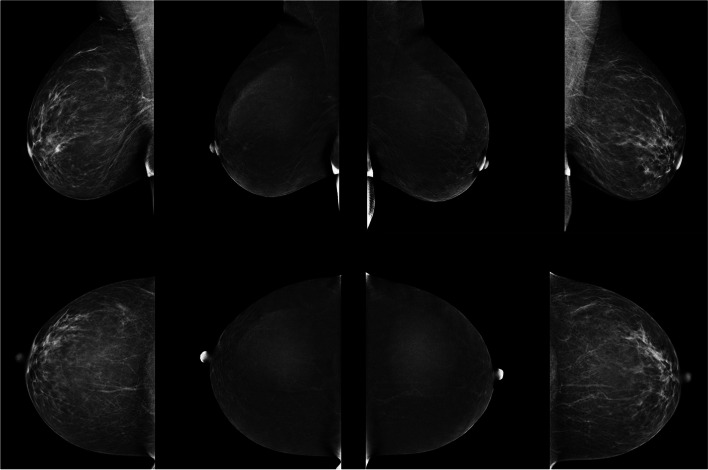
Representative normal CEM study with standard 8 views showing the low energy (LE) and recombined images (RI) in a patient with no abnormal enhancement in either breast

LE and RI are immediately reviewed by a radiologist present in the mammography suite at the time of examination for the presence of abnormal contrast enhancement (Fig. 2). If needed, additional diagnostic views, such as true lateral, exaggerated or spot compression views can be obtained, though must be completed within the 10-minute time frame. If magnification views are needed, those can be acquired outside of CEM mode and beyond the 10-minute window. Targeted ultrasound to the area(s) of abnormal enhancement may follow when indicated, resulting in an increased yield of lesion detection and improved characterization. The results of the study are immediately reviewed with the patient, alleviating a gamut of emotional and psychological stresses associated with prolonged wait times.

## Regulatory compliance

As with any contrast administration, assessment for risks and benefits to each individual patient must be made prior to performing CEM. This is achieved by following the American College of Radiology Contrast Manual guidelines [15]. The main risks related to contrast administration involve an allergic-like reaction, contrast-induced nephrotoxicity in addition to other non-allergic adverse reactions.

Additionally, there are state regulations regarding administration of contrast, which may be performed by a radiologist, radiology technologist, or nurse [15]. Administration should be performed via an adequately prepared power injector apparatus.

Radiation dose is an omnipresent concern across modalities with ionizing radiation and cannot be overlooked in evaluation of CEM. For CEM, some studies estimate radiation doses between 20 and 45% higher than traditional 2D mammography [9, 20–24], though some studies have estimated a smaller difference in dose. Continued equipment improvements, modifications and upgrades ultimately result in decreased radiation doses.

Similar to traditional mammography practices, daily and weekly quality control practices must be performed for CEM systems to ensure consistency and avoid artifacts which may be related to calibration [25].

## Current indications

Table 1 summarizes the most common indications for CEM at this time.

**Table 1:** Current Indications for CEM

High-risk screening (not an FDA approved indication but under investigation)	
Supplementation to screening mammography in heterogeneously dense and extremely dense breast parenchyma in accordance with local state regulations, usually in the setting of dense breast clinic	
Further assessment of inconclusive findings on diagnostic workup	
Assessment of palpable abnormality with negative prior workup	
Staging of known breast cancer, particularly in patients with contraindications to MRI	
Assessment of response to chemotherapy, especially in patients with contraindications to MRI	

While prior studies have predominantly been performed investigating the use of CEM as further diagnostic evaluation and utilization in newly diagnosed cancer [26–28], a recent study at Memorial Sloan Kettering Cancer Center has investigated the use of CEM as a primary screening tool [29] with optimistic results, however, screening is not an FDA-approved indication at the present time.

## Normal background CEM findings

**Fig. 3:**
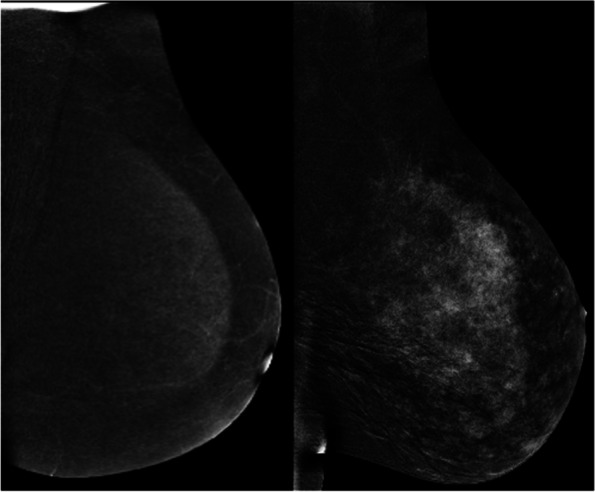
Contrasting examples of a patient with scattered fibroglandular breast tissue who exhibits minimal BPE on RI (left) versus a patient with extremely dense breast tissue who exhibits marked BPE on RI (right)

Similar to contrast-enhanced breast MRI, there is a range of background parenchymal enhancement (BPE) which may be categorized as 1) None/Minimal, 2) Mild, 3) Moderate, or 4) Marked (Fig. 3). Most patients tend to exhibit minimal or mild background as demonstrated in a recent study by Berg WA et al. [13], with 95% of 1000 patients in a preliminary study performed at our own institution also falling into these categories. Compared to breast MRI, BPE tends to be far less on CEM. Only 1.3% of over 2000 cases studied at our institution had marked BPE with a strong correlation to having dense breasts (heterogeneously dense or extremely dense). Additionally, premenopausal women have been found to be significantly more likely to have marked BPE compared to postmenopausal women. There is a suggestion that BPE on CEM correlates with breast cancer risk [30] which may be an important quantification for use in risk assessment tools.

## Benign CEM findings

### Cysts

**Fig. 4:**
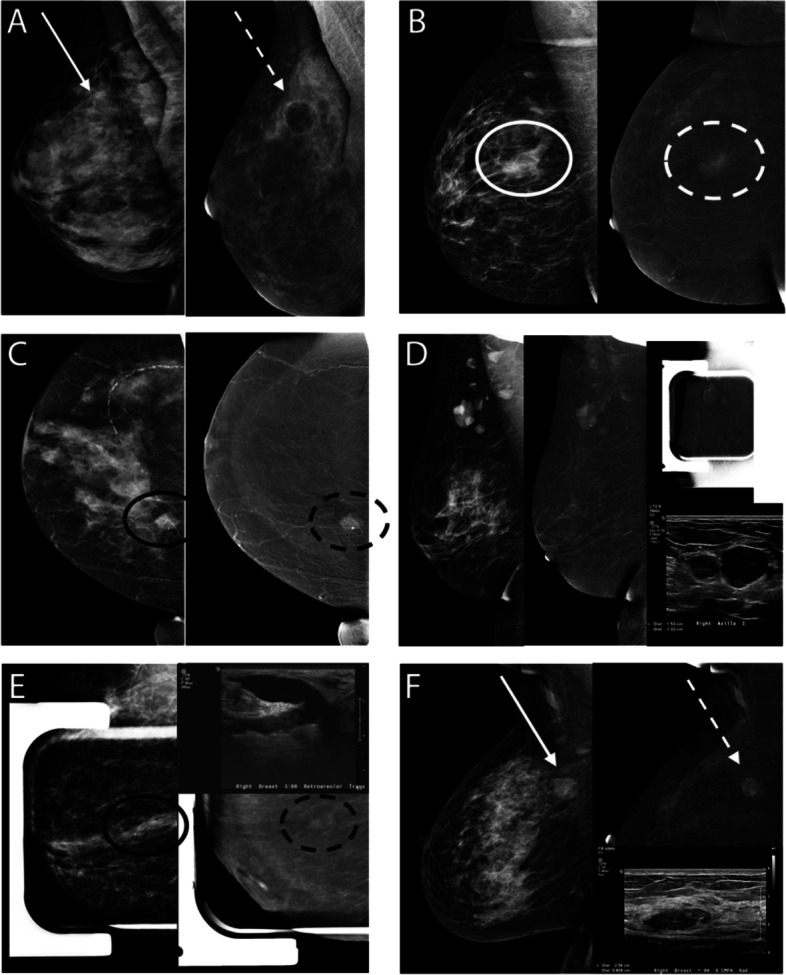
Examples of Common Benign Pathologies seen on CEM. **A** Typical CEM appearance of cyst on LE (solid white arrow) and RI CEM (dash white arrow) with negative internal enhancement and thin rim enhancement also known as the “eclipse” sign. **B** Region of non-mass enhancement (dash circle) with irregular margins on LE (solid circle) corresponds to biopsy proven localized fibrocystic change. **C** Call back from screening mammogram for further characterization of mass in the medial breast. A 1.9 cm lobulated mass with associated solitary coarse calcification (solid black circle) demonstrates homogeneous enhancement (dash black circle) and corresponds to biopsy proven fibroepithelial lesion compatible with fibroadenoma. **D** Call back from screening for enlarging right axillary lymph nodes on screening mammography. CEM was performed showing enhancing circumscribed masses in the right axilla. Ultrasound revealed abnormal lymph nodes with thickened cortices. Biopsy subsequently revealed reactive changes within a biopsied lymph node. **E** Abnormal findings on the baseline screening mammogram, CEM recommended for further evaluation. Spot compression of retroareolar focal asymmetry (solid black circle) reveals focus of enhancement (dash black circle) at the end of the linear negative enhancement, representing a dilated duct. Corresponding ultrasound revealed intraductal hypoechoic mass, subsequently biopsy proven as intraductal papilloma. **F** Call back from screening mammography for bilateral abnormalities. Round mass with indistinct borders in upper inner right breast, posterior depth (solid white arrow) on LE images demonstrates enhancement on RI CEM (dash white arrow). Ultrasound of the right breast at 1:00, 8 cm from nipple, revealed an oval parallel hypoechoic circumscribed mass, subsequently biopsy proven as PASH

Simple, inflamed, and complicated cysts are commonly appreciated within the breast. A simple cyst of the breast presents on the mammogram as a low to equal density circumscribed or partially obscured mass. On RI, cysts are generally negatively enhancing lesions with thin or imperceptible enhancement of the wall (Fig. 4A), previously characterized as an “eclipse” sign [31]. However, if there is acute or subacute inflammation of a cyst, there may be an enhancing mildly thickened rim, similar to characteristic rim-enhancement on MRI of the breast. Confirmation may be performed by subsequent ultrasound following CEM, guiding cyst localization, which may be important given overlapping appearances with certain centrally necrotic malignancies including triple negative invasive ductal carcinomas.

### Fibrocystic changes

Fibrocystic changes have a large spectrum of appearances on mammography as well as other imaging modalities [32]. In our institution’s experience, a localized area of fibrocystic change may be seen as a region of non-mass enhancement (Fig. 4B). Occasionally, we have observed diffuse bilateral BPE containing variable sizes of negative enhancement corresponding to parenchymal cysts. If the finding is asymmetric and localized to one side/quadrant, this nonspecific enhancement may prompt biopsy for definitive diagnosis, especially if corresponding to an area of palpable concern.

### Fibroadenomas

Fibroadenomas may be seen mammographically as circumscribed round or oval masses, with or without associated characteristic popcorn calcifications. Often, these are T2 hyperintense masses which demonstrate variable enhancement on MRI, sometimes with characteristic thin internal, non-enhancing septations. During CEM imaging, fibroadenomas may demonstrate variable degrees of enhancement (Fig. 4C) while others do not enhance at all due internal hyalinization, akin to MRI findings. Evaluation of LE images on CEM combined with RI leads to increased detection of the enhancing finding on subsequent ultrasound which, in turn, allows for further characterization of these benign masses eliminating the need for biopsy.

### Lymph nodes

While intramammary lymph nodes most commonly occur in the upper outer breast quadrant, they can be found anywhere in the breast. Generally, lymph nodes are circumscribed reniform masses on LE images, typically with demonstration of characteristic fatty hila and variable degree of enhancement on subtraction CEM images (Fig. 4D); ultrasound allows definitive characterization.

### Intraductal papillomas

Solitary intraductal papillomas often have non-specific mammographic appearances as they may range from an occult appearance, particularly given less optimal compression and increased breast density in the retroareolar region, to round or oval masses with circumscribed margins. On RI CEM, papillomas may present as focus of enhancement or as a discrete mass (Fig. 4E) especially if greater than 5 mm in length with or without accompanying areas of focal duct dilatation on LE images. Further imaging with ultrasound may demonstrate an intraductal mass within a dilated duct, a complex cystic and solid mass, or a solid hypoechoic mass [32]. Given the non-specific CEM and ultrasound findings, biopsy is required for definitive diagnosis. However, when comparing CEM and MRI for papillomas specifically, a recent study has shown that MRI has a significantly higher sensitivity than CEM for diagnosis of intraductal papilloma no matter the size of the lesion [33].

### PASH

Pseudoangiomatous stromal hyperplasia (PASH), a benign myofibroblastic proliferation process, may present mammographically as a developing parenchymal asymmetry or less commonly as a mass. On RI CEM, variable enhancement may be seen corresponding to parenchymal asymmetry or mass (Fig. 4F).

### Phyllodes tumor

Phyllodes tumors are solitary, unilateral tumors classified as benign, borderline, or malignant depending on histology, typically requiring full excision. While these represent less than 1% of all breast tumors [32], the overlapping appearance of phyllodes tumors with fibroadenomas is commonly encountered. Similar to fibroadenomas, phyllodes tumors may present as masses on mammography. On RI CEM, phyllodes tumors are seen as enhancing circumscribed masses, with variable degree of enhancement (Fig. 7A). Again, similar to most masses with non-specific imaging appearance/pattern of enhancement, final diagnosis must be obtained with tissue sampling.

## Malignant CEM findings

Since tumor angiogenesis corresponds to enhancement seen both on CEM as well as MRI, there is a possible role for CEM to be used in staging as well as assessment of treatment response, particularly in patients who have contraindications to MRI, with recent studies supporting this concept [9, 19, 34]. CEM has been shown to be accurate in determining tumor size compared to the gold standard of pathology in addition to being more precise than traditional mammography and ultrasound [34]. Particularly for invasive cancer, a recent study showed CEM sensitivity to be particularly high, with a 98% detection rate [10].

### Ductal carcinoma in situ (DCIS)

**Fig. 5:**
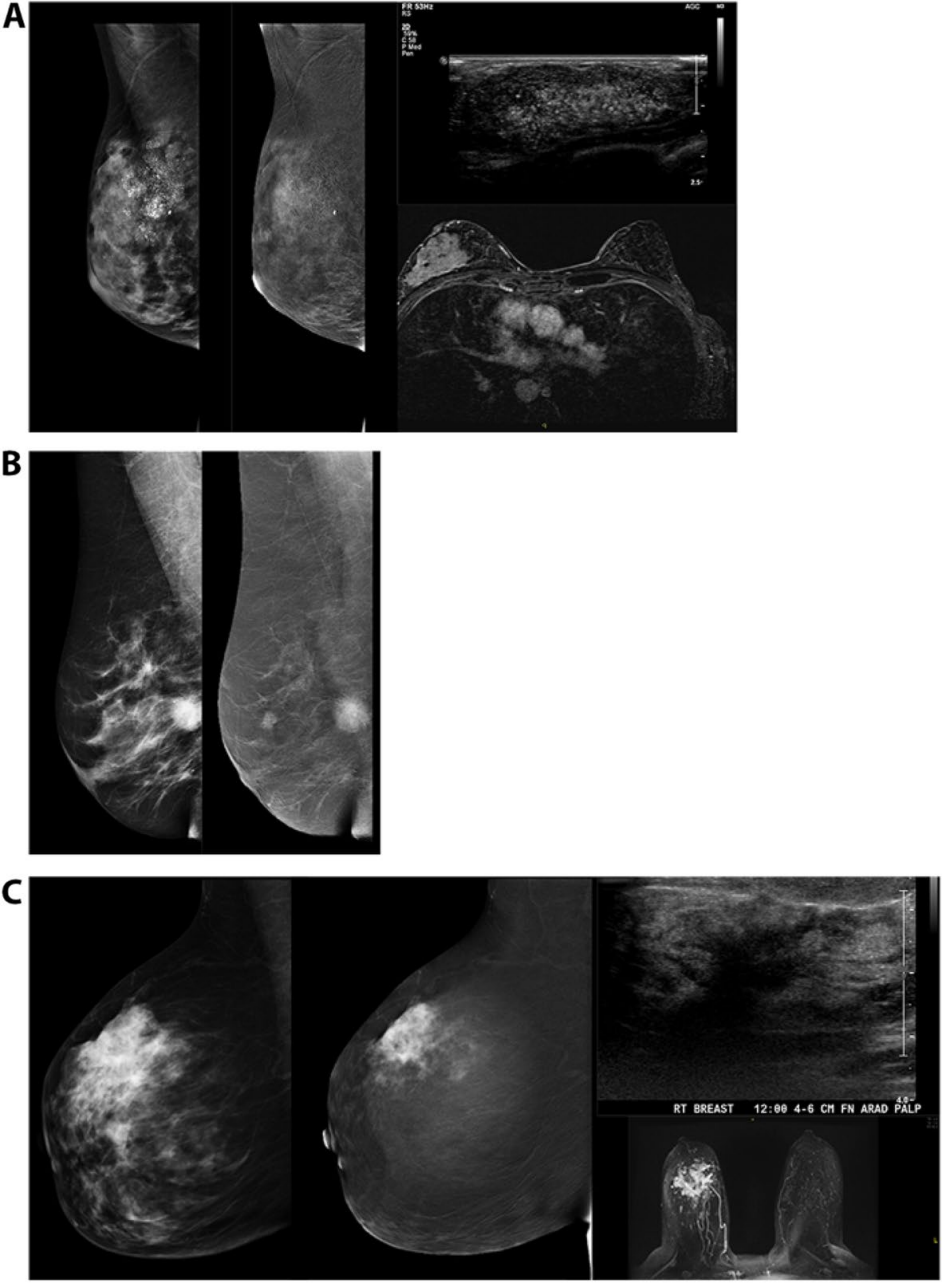
Malignant Pathologies (DCIS, IDC, ILC). A) 42-year-old woman presented with new palpable lump. Extensive pleomorphic microcalcifications throughout upper outer breast span at least 8 cm on LE images with non-mass enhancement in region of microcalcifications on RI. Ultrasound performed in this area revealed hypoechoic mass with numerous echogenic foci, subsequently biopsied revealing DCIS. **A** preoperative MRI revealed a large area of non-mass enhancement in the same distribution. Upon mastectomy, the pathology was upgraded to invasive ductal carcinoma. **B** 47-year-old woman called back from screening for new mass. Round spiculated 2.1 cm mass in the inferior central breast at posterior depth on LE images demonstrates avid enhancement on RI in addition to demonstrating smaller satellite masses in the upper central breast. After subsequent biopsy and mastectomy, findings on pathology were compatible with multicentric invasive ductal carcinoma. **C** 44-year-old woman presented with palpable lump in R breast. On CEM, a large high density spiculated mass at 12:00 in the mid breast on LE images is associated with avid enhancement on RI; US confirmed spiculated shadowing mass. MRI showed findings similar to CEM. Final diagnosis: invasive lobular carcinoma with associated LCIS

DCIS is a heterogeneous disease with variable range in grade, most commonly presenting in various calcification patterns. While less common, DCIS can produce a mass or architectural distortion. Sometimes DCIS is mammographically occult, presenting without associated microcalcifications, and MRI may demonstrate clustered ring or linear non-mass enhancement to allow diagnosis. In our experience with CEM, DCIS may similarly manifest as areas of non-mass enhancement with or without microcalcifications (Fig. 5A). In general, the higher the grade of DCIS, the more intense is the associated parenchymal enhancement. Additionally, the larger the area of involvement with DCIS, the more evident is the disease process, particularly in combination of LE and RI evaluation. If the group of microcalcifications is less than 5 mm in length, there may not be perceived focal enhancement. In these cases, morphology of the microcalcifications on the accompanying LE imaging will guide management.

### Invasive ductal carcinoma

Invasive ductal carcinoma (IDC) has a spectrum of appearances although most commonly presents as a round or spiculated mass on LE images. On CEM, IDC typically enhances on the RI. The degree of enhancement is based on tumor grade and size, with larger, more aggressive tumors demonstrating stronger enhancement (Fig. 5B). Compared to mammography alone however, CEM has the ability, particularly within dense breast tissue, to identify mammographically occult findings. Similar to MRI, IDC may present as non-mass enhancement, both in segmental and regional patterns on RI.

### Invasive lobular carcinoma

Invasive lobular carcinoma (ILC) often presents less discreetly on mammography, sometimes with diffuse breast changes, distortion, or asymmetry. Enhancement patterns may vary more extensively with ILC due to differences in angiogenesis relative to IDC. Given the known propensity of ILC to grow in a lepidic pattern, it may be perceived as asymmetric focal non-mass enhancement on RI (Fig. 5C), best seen only on one projection, again highlighting the importance of technique, the need for evaluation of areas of enhancement between RI in addition to the need to maintain a high degree of suspicion for lesion detection coupled with evaluation of LE images. If ILC is mass-forming, various degrees of enhancement may be seen in association with the mass on RI, similar to IDC, and depend on the size of the tumor.

### Associated features of cancer

**Fig. 6:**
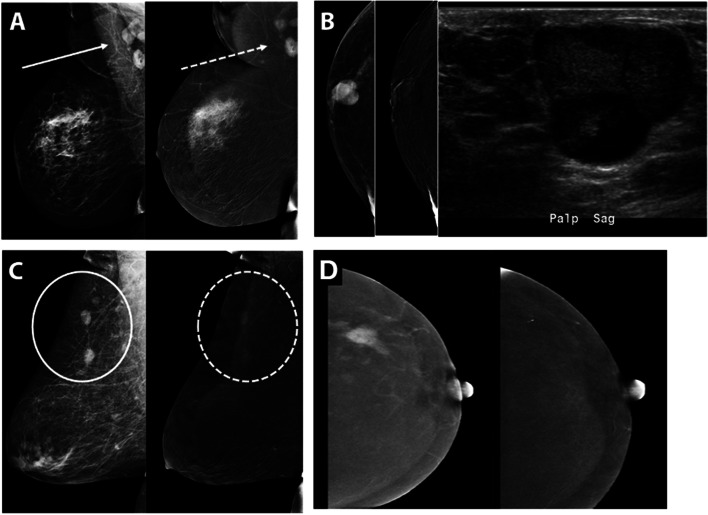
Associated Features of Malignancy and Other Entities. **A** Patient with multicentric breast cancer with enhancing metastatic disease to the right axilla (solid arrow corresponding to axillary lymph nodes on LE, dash arrow corresponding to enhancing axillary lymph nodes on RI). **B** 61-year-old man presented with palpable enhancing subareolar mass. Ultrasound correlate revealed a circumscribed hypoechoic mass with posterior enhancement, subsequently biopsied to reveal invasive ductal carcinoma with papillary features, for which a mastectomy was performed with curative intent. **C** 59-year-old woman with history of lymphoma presented with multiple oval enhancing masses in the breast (solid circle on LE, dash circle on RI) compatible with biopsy proven diffuse B cell lymphoma. **D** 56-year-old woman with known infiltrating ductal carcinoma, shown before and after neoadjuvant chemotherapy RI CEM pre-treatment image shows an enhancing spiculated mass with resolution of enhancement and mass on post-treatment RI (clip indicates an area of biopsy-proven malignancy)

As an alternative for patients who may not be able to undergo MRI during staging of breast cancer, CEM has been shown to be helpful in characterizing the extent of disease and evaluating for other occult malignant lesions with an added advantage of significantly decreased background enhancement in most patients, increasing specificity. Some features noted on CEM include identifying abnormal and potentially metastatic lymph nodes on LE and RI (Fig. 6A), nipple involvement, multifocal or multicentric, and contralateral disease. CEM has a limited role in assessment of posterior tumor extent or chest wall involvement due to limitations in patient positioning and is unable to assess internal mammary lymph nodes. While predominantly utilized in evaluation of breast disease in women, CEM has been successfully utilized in evaluation of abnormal findings in men as well (Fig. 6B). Occasionally, other malignancies will present with findings of breast masses on mammography and may be evaluated with CEM (Fig. 6C). CEM has also been successful in assessing treatment response appropriately demonstrating patients with complete and incomplete imaging response to therapy (Fig. 6D).

### High risk lesions

**Fig. 7:**
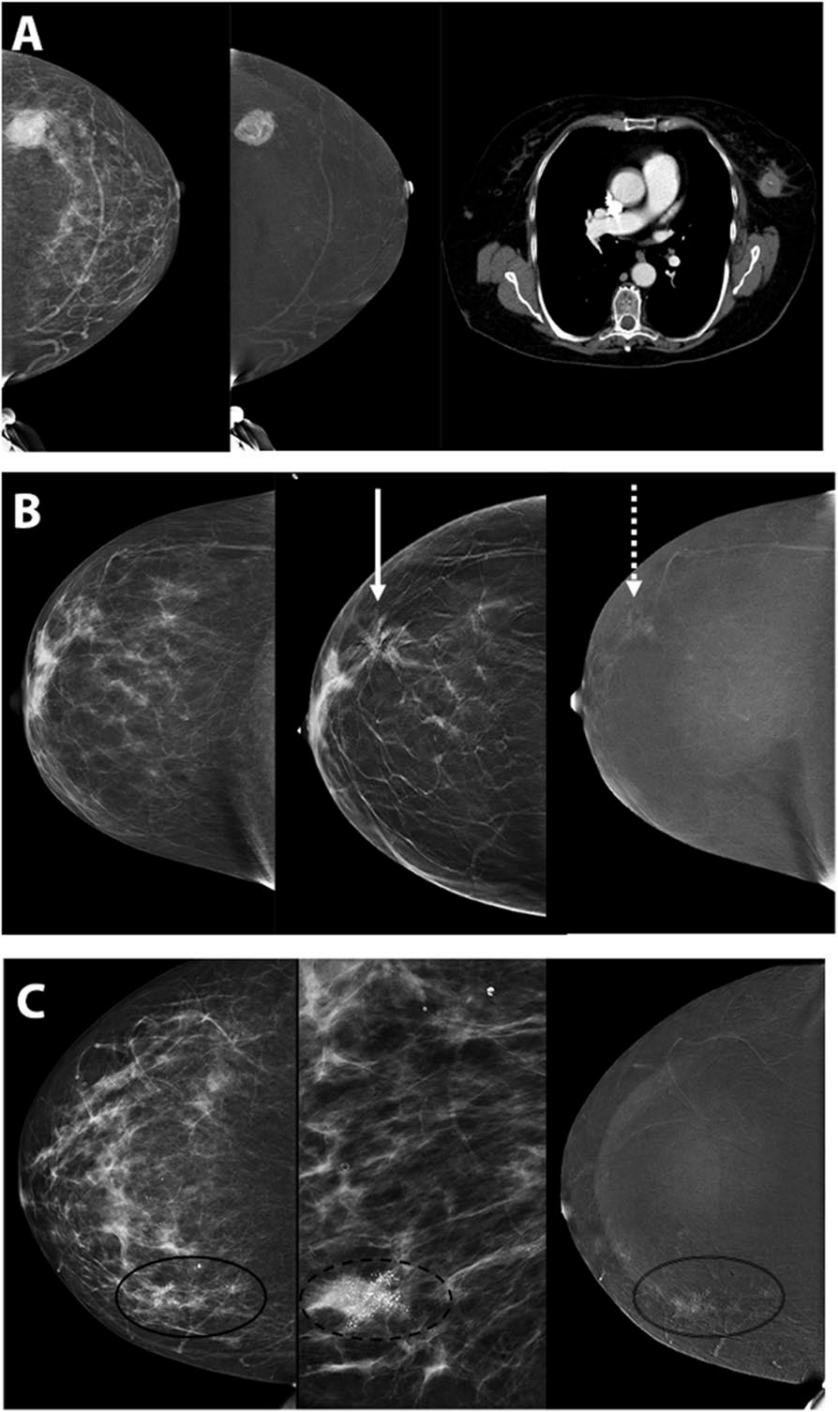
High Risk Lesions or Borderline. **A** 76-year-old woman presented with new hyperdense mass in left upper outer breast on LE images, with avid enhancement and central necrosis on RI CEM. Patient unable to undergo MRI due to pacemaker but subsequent staging CT chest image shows a corresponding mass. Patient underwent left mastectomy which revealed malignant phyllodes tumor. **B** 40-year-old woman presenting for baseline screening mammogram, called back for architectural distortion. On CEM diagnostic workup, there is persistence of the architectural distortion (solid arrow) on LE images with minimal central enhancement (dash arrow). Ultrasound (not pictured) revealed a subtle area of distortion. Subsequent biopsy was compatible with radial scar, atypical ductal hyperplasia, intraductal papilloma. **C** Patient presented with suspicious microcalcifications (solid circle on LE image, dash circle on magnified view, and double line circle corresponding to non-mass enhancement on RI)

High risk lesions including atypical ductal hyperplasia (ADH), lobular neoplasias such as lobular carcinoma in situ (LCIS) and atypical lobular hyperplasia (ALH), papillomas, radial scars (Fig. 7B), mucinous lesions, and flat epithelial atypias, are frequently encountered diagnoses. Features on CEM often overlap with other benign or malignant entities, noting an example of LCIS shown (Fig. 7C) with non-mass enhancement in association with suspicious calcifications. Careful assessment of LE images is required: lack of enhancement on RI does not alleviate the need to biopsy suspicious findings on LE and lack of or perceived minimal enhancement should not sway management.

## Limitations

### False positive CEM findings

In addition to aforementioned benign entities, a couple specific scenarios may also lead to false positive CEM enhancement.

#### Enhancement of skin lesion

**Fig. 8:**
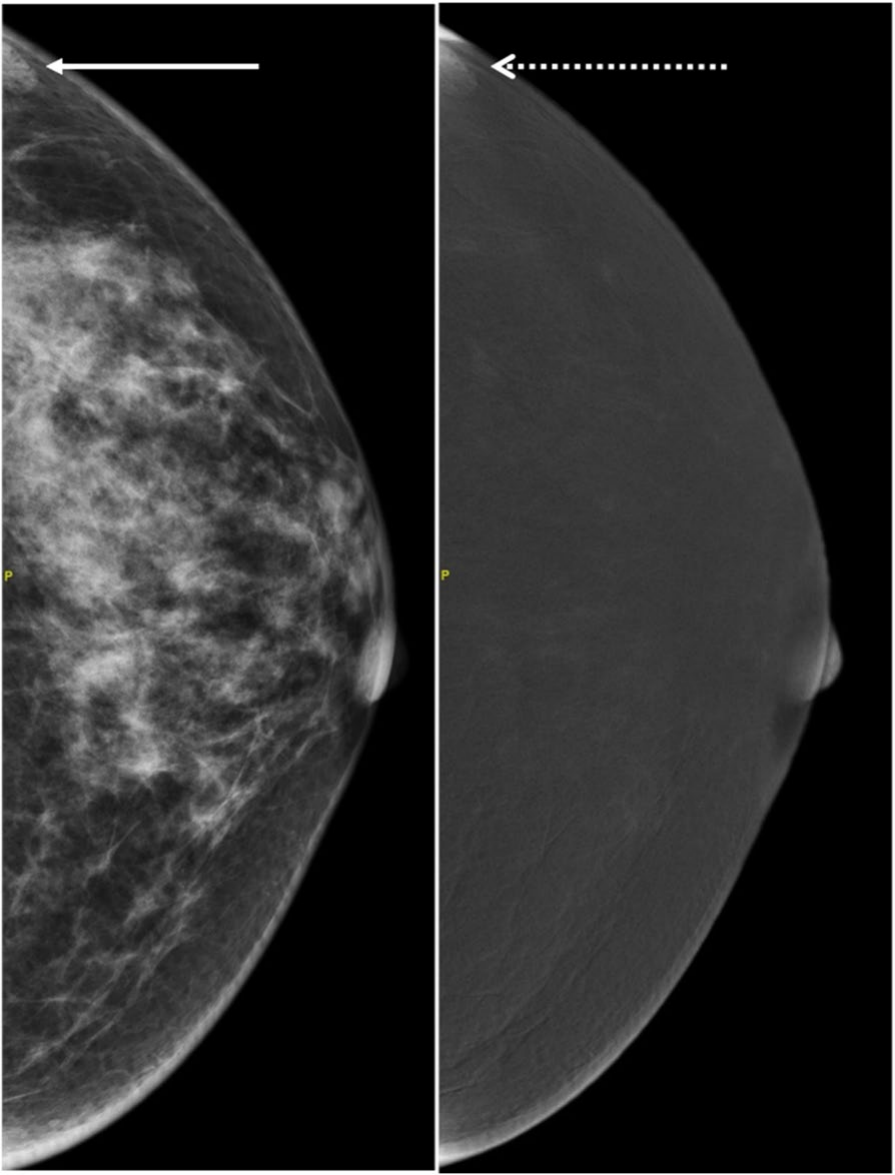
58-year-old woman presented for diagnostic work up for abnormal findings in both breasts. LE image shows an asymmetry (solid arrow) in the posterior far lateral left breast with corresponding enhancement on the RI (dash arrow). Upon physical exam at the time of ultrasound, this area of enhancement correlated to a skin lesion, seborrheic keratosis

Some skin lesions, such as moles or seborrheic keratosis can enhance (Fig. 8). On a CEM LE images, dermal location of the finding may not be immediately evident. It may require physical examination and/or correlation with tomosynthesis.

#### Inflammation or infection

**Fig. 9:**
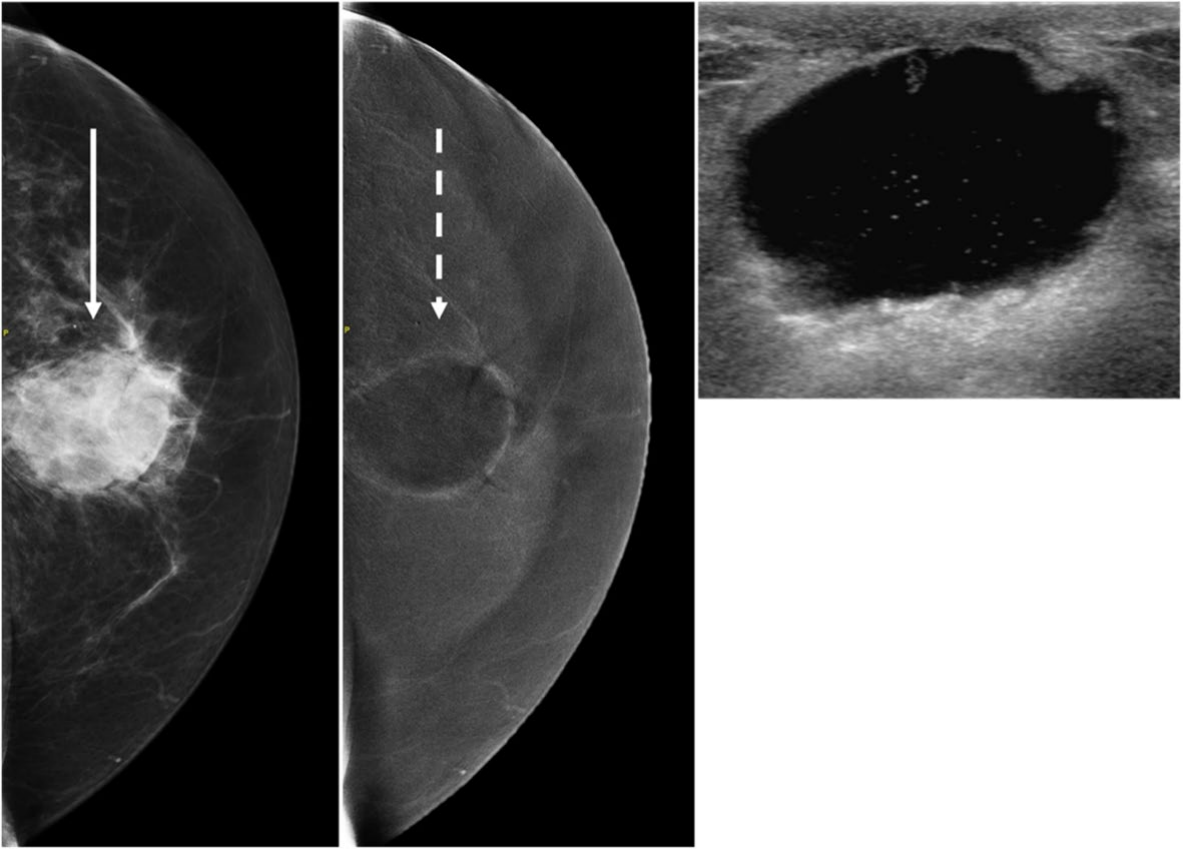
63-year-old woman with left breast palpable abnormality 7 months post excisional biopsy for atypia. CEM shows a dense round mass (solid arrow) on LE image in the central left breast with associated rim enhancement on RI (dash arrow). On subsequent ultrasound, a fluid collection with mildly thickened wall in the area of previous excision corresponds to the mammographic finding, supporting benign etiology of postoperative seroma

In the post-procedure/postoperative setting, due to an underlying seroma, hematoma, infection or fat necrosis, there may be associated, predominantly peripheral enhancement on CEM (Fig. 9). However, relatively thin rim of enhancement on RI due to inflammation should be distinguished from irregular, nodular or mass-like enhancement which may represent true positive enhancement of residual or recurrent disease.

### False negative CEM findings

Potential reasons CEM can lead to false negative diagnoses are categorized into three main scenarios: (1) lack of enhancement due to small size and/or low grade histology of ductal carcinoma in situ or invasive breast cancer (true false negative), (2) missed abnormal enhancement due to marked BPE or misinterpretation of true enhancement as BPE and (3) limitations of the technique (e.g. the abnormal finding is not included in the image due to its location).

**Fig. 10:**
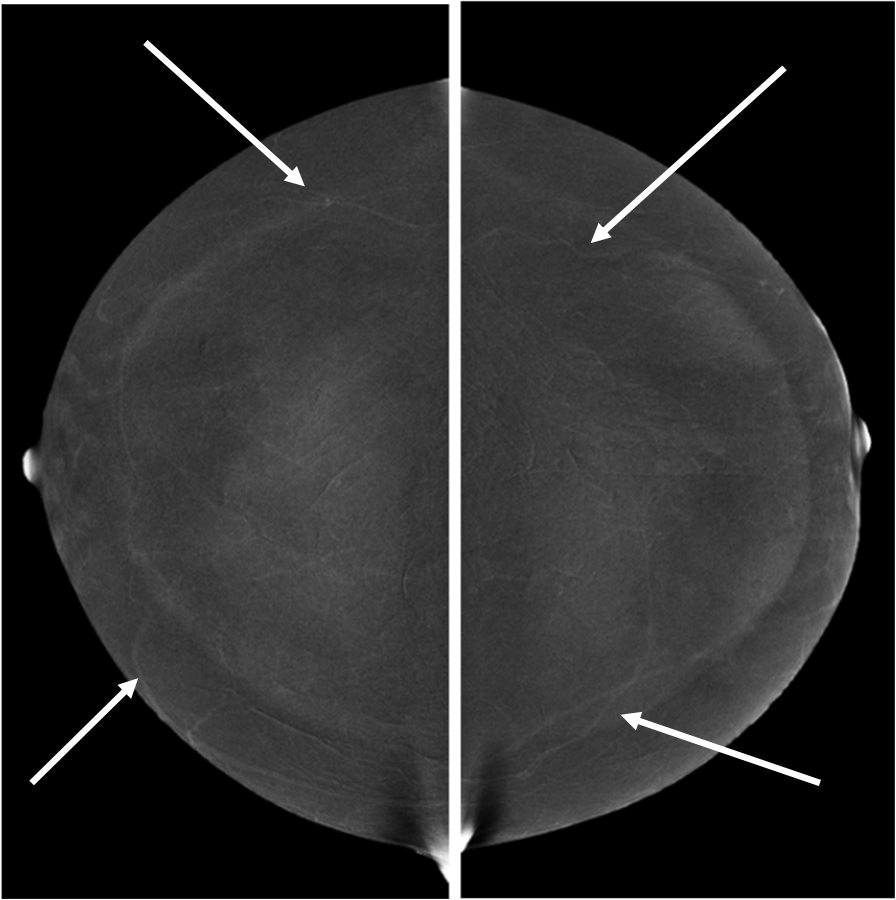
Bilateral CEM RI in CC projections demonstrate contrast within breast vessels (white arrows) supporting adequate contrast bolus

CEM relies on the principle of tumor angiogenesis, vessel immaturity and increased permeability, resulting in diffusion of contrast into the tumor, subsequently manifesting as contrast enhancement [3, 35, 36]. To ensure technical adequacy, similar to evaluation of breast MRI, assessment of the CEM images must be made for adequacy of contrast administration. Visualization of contrast in breast vessels is a good indicator of an adequate contrast bolus (Fig. 10).

Lack of contrast enhancement on CEM RI might be caused by inadequate contrast bolus due to many factors including small caliber of the injection catheter, inadequate rate of contrast administration, contrast extravasation, peripheral/central vessel occlusion or impaired cardiovascular function. In addition, motion may further limit lesion detection and characterization, especially if the cancer is small.

While MRI is overall a very sensitive test for breast cancer detection, false negative cases have also been reported [37–40]. In a non-randomized prospective multicenter study [38], false negative rates for MRI with respect to breast cancer detection were reported as 22% (22/97 breast cancers in 2157 women). In six patients (27% of the false negative cases), breast cancer was missed or misinterpreted due to small lesion size (13% of false negative cases), extensive diffuse contrast enhancement of breast parenchyma (9%), and technically inadequate examination (4.5%). 43% of such false negative cases (9/21) were pure DCIS or DCIS with invasive foci. 90% of false negative DCIS (8/9) had no enhancement on MRI.

#### Lack of enhancement due to small size

**Fig. 11:**
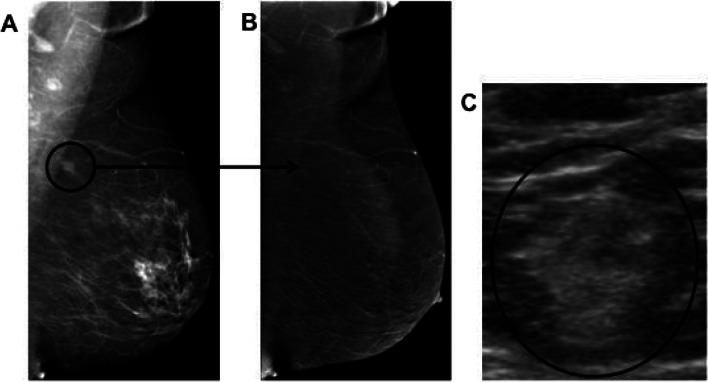
Example of lack of enhancement due to small size. Patient was referred from an outside institution for abnormal screening mammogram. **A** CEM shows a 1.1 cm spiculated mass (black circle) on the LE image in the posterior left upper breast, axillary tail region, only seen on the MLO view. **B** No enhancement is seen on RI. **C** Subsequent ultrasound revealed an indistinct 1.2 cm echogenic mass (black circle) which correlated with the mammogram finding. Ultrasound guided biopsy confirmed invasive ductal carcinoma

Similar to these observations with MRI, small cancer size possibly coupled with low grade histology and ductal carcinoma in situ (DCIS) with no associated mass are two reasons for the possible lack of enhancement of breast cancer on CEM. Regarding small size, tumor angiogenesis is incited as cancer grows typically beyond 0.3 cm and thus, like MRI [40], it can be extrapolated that false negatives may be seen with very small invasive carcinomas on CEM (Fig. 11).

#### Lack of enhancement in ductal carcinoma in situ

**Fig. 12:**
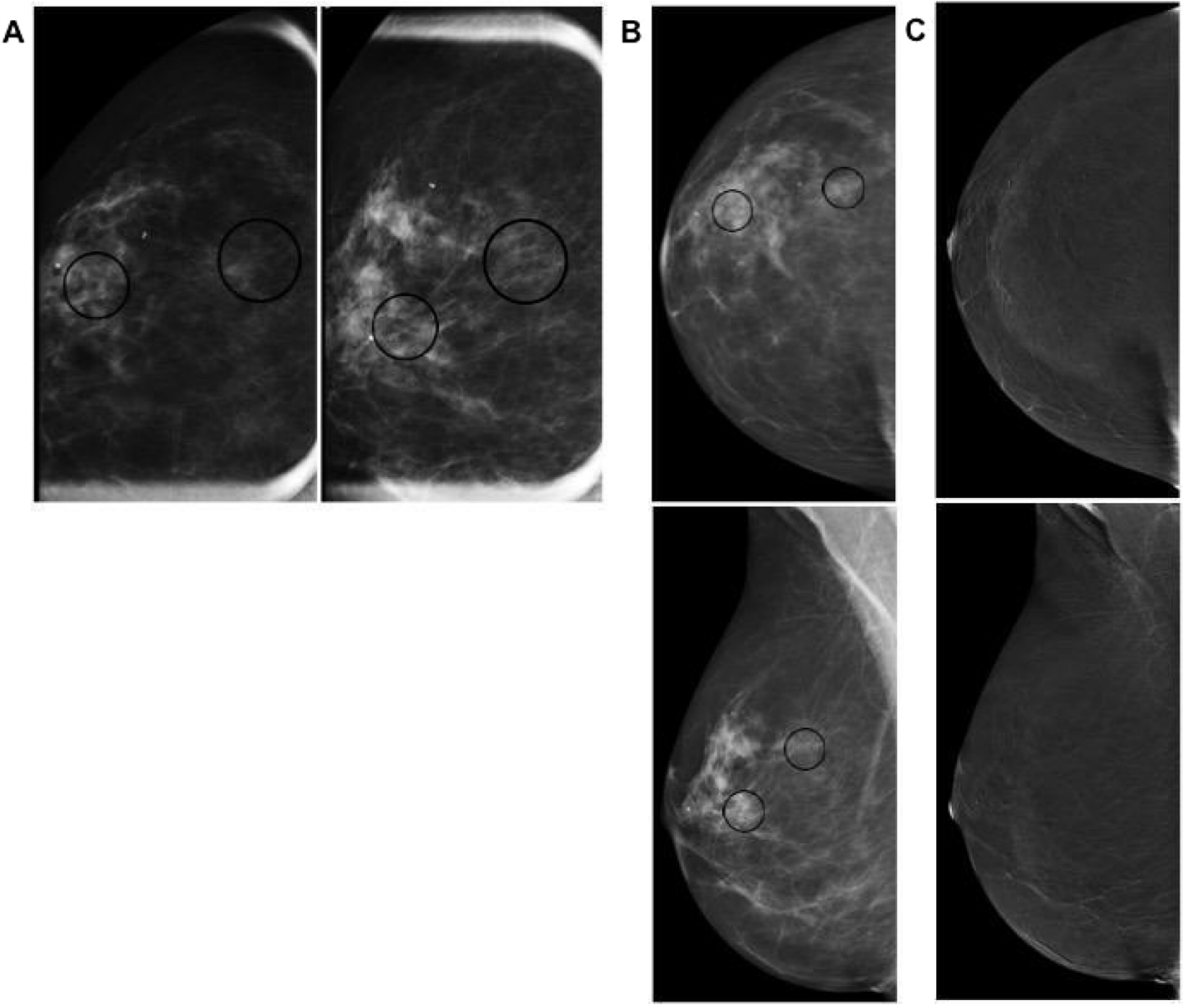
64-year-old female was called back from screening. (**A**) Spot magnification views in the CC and ML projections show two groups of suspicious microcalcifications (black circles), one of which is associated with a vague focal asymmetry which persists on spot compression (**B**); same day CEM study again shows the two groups of microcalcifications (black circles) on the LE images (**C**) but with no suspicious enhancement on RI. A stereotactic needle biopsy was performed confirming low grade DCIS. This case illustrates how CEM can protect sensitivity in the example of DCIS on LE mammogram by identifying the morphologic abnormality of microcalcifications before assessing enhancement on subtraction mammogram of CEM (no enhancement is identified in this case)

Lack of enhancement is more common in DCIS because the degree of angiogenesis is lower in DCIS than in invasive carcinomas [40]. Unlike MRI, CEM maintains sensitivity and specificity of diagnostic mammography for detecting a non-enhancing small invasive cancer or DCIS by detecting morphologic abnormalities such as focal asymmetry, distortion or microcalcifications [41, 42]. DCIS, presenting as microcalcifications as the only manifestation, can be identified on the LE image of CEM alone (Fig. 12), with additional evaluation by magnification views, as needed. This accounts for approximately 10% of cancers presenting with microcalcifications. CEM, therefore has the added value to identify DCIS with or without lesion enhancement. Stereotactic-guided needle core biopsy is usually subsequently used for a tissue diagnosis in such cases.

#### Missed abnormal enhancement due to marked background parenchymal enhancement (BPE)

An enhancing cancer may be missed if there is significant BPE or when there is asymmetric BPE. Just as in breast MRI, the level of BPE is reported on all CEM studies in order to convey to the reader the level of sensitivity. In contrast to breast MRI, marked BPE appears to be less prevalent on CEM studies based on our experience as well as Berg et al. 2021 [13].

#### Misinterpretation of true enhancement as background enhancement

Misinterpreted enhancement relates to misinterpreting cancer enhancement as benign, which has been described on MRI [40] and may be encountered on CEM. Misinterpretation may be due to the pattern of enhancement on RI (e.g. non-mass enhancement mimics asymmetric BPE). This could be a particular challenge when cancer enhancement presents as non-mass enhancement with a relatively lower degree of enhancement intensity, potentially coupled with similar enhancement to that of BPE.

#### Inappropriate technique

Not unique to CEM, imaging with inappropriate technique, can lead to “missing” the cancer. Enhancement may be missed if it is in a portion of the breast that is not in the imaging field (e.g., posterior breast mass on a study where there is not enough posterior breast tissue included on the image) [41]. Additionally, there are artifacts inherent to CEM [43] which may obscure findings, further highlighting importance of proper technique.

#### Modifications to diagnostic protocol to protect sensitivity of CEM

Several factors can lead to a false-negative CEM study as discussed above. Thoroughly assessing abnormal findings on LE is an important first step to protect the sensitivity of CEM in detecting high risk lesions, DCIS and/or small low-grade IDC. Adding breast ultrasound with CEM as an adjunctive diagnostic test will also increase sensitivity for lesion detection and characterization [5, 18] helping avoid unnecessary biopsies and additional short-term follow up studies. If there is a palpable abnormality without correlate on CEM/US, abnormal findings on LE images or clinical suspicion warrants, MRI should follow to look for an occult cancer.

Lastly, enhancing CEM findings may be challenging to biopsy without ultrasound correlate, necessitating the need for MRI for visualization and subsequent sampling. New CEM equipment allows biopsy capabilities, some in combination with tomosynthesis.

## Conclusion

CEM continues to grow in utilization at practices around the world. Although it has been studied most frequently in the setting of malignancy and assessing extent of disease, there is increased utilization for variable scenarios, including in the screening setting. While we continue to study and characterize both benign and malignant pathologies encountered in CEM, additional studies will elucidate more specific CEM findings. The application of adapted BIRADS lexicon terms to CEM findings continues to evolve and improve interpretation and standardized reporting.

## Data Availability

Not applicable.

## Abbreviations

CEM: Contrast-enhanced mammography
FFDM: Full field digital mammography
LE: Low energy
HE: High energy
RI: Recombined images
BPE: Background parenchymal enhancement
PASH: Pseudoangiomatous stromal hyperplasia
DCIS: Ductal carcinoma in situ
IDC: Invasive ductal carcinoma
ILC: Invasive lobular carcinoma
ADH: Atypical ductal hyperplasia
LCIS: Lobular carcinoma in situ
ALH: Atypical lobular hyperplasia
